# A Tale of Two Scopes: Comparative Evaluation of Tuoren® and King Vision® Videolaryngoscopes in Patients Undergoing General Anesthesia

**DOI:** 10.7759/cureus.113671

**Published:** 2026-07-30

**Authors:** Vatsala, Koshy Varghese, Amlan Swain, Seelora Sahu, Deb Sanjay Nag, Abhishek Karthik

**Affiliations:** 1 Anaesthesiology, Tata Main Hospital, Jamshedpur, IND; 2 Anaesthesiology, Manipal Tata Medical College, Jamshedpur, IND

**Keywords:** airway management, anesthesia, ease of intubation, intubation time, king vision, tuoren, videolaryngoscope

## Abstract

Background: Laryngoscopy remains a cornerstone of airway management in anesthesiology. With advancements in technology, videolaryngoscopes have improved visualization and intubation success rates. Among these, the Tuoren® videolaryngoscope (Tuoren Medical Group, Henan, China) and the King Vision® videolaryngoscope (Ambu A/S, Ballerup, Denmark) have been widely used, although direct clinical comparisons between the two devices remain limited. This research sought to assess and compare ease of intubation, glottic visualization, time efficiency, and success rates between the Tuoren® and King Vision® videolaryngoscopes.

Methods: A prospective, randomized, single-blind study was conducted on 98 adult patients with American Society of Anesthesiologists (ASA) physical status I-III undergoing elective surgeries requiring general anesthesia. The participants were allocated into two groups of 49 each: Group I was intubated using the Tuoren® device and Group II using the King Vision® device, after standard induction and neuromuscular blockade. The recorded parameters were intubation time, number of attempts, ease of intubation score, glottic visualization (modified Cormack-Lehane and Percentage of Glottic Opening (POGO) scores), and hemodynamic responses. Statistical analysis was performed using the IBM SPSS Statistics software for Windows, version 21.0 (IBM Corp., Armonk, NY, USA).

Results: Patient demographics and baseline airway assessments were comparable between the groups. The mean intubation time was significantly shorter with the Tuoren® videolaryngoscope compared to the King Vision® videolaryngoscope (64.6 ± 28.8 s vs. 79.6 ± 13.2 s, p < 0.001). The Tuoren® group demonstrated a higher median ease of intubation score (3 (IQR 3-4) vs. 3 (IQR 3-3), p = 0.0011). Although King Vision® provided superior glottic views (p = 0.002), this did not translate to easier or faster intubation. Hemodynamic parameters and complication rates were similar between the two groups.

Conclusion: Both Tuoren® and King Vision® videolaryngoscopes are effective for tracheal intubation under general anesthesia. While the King Vision® videolaryngoscope offers a marginally better glottic view, the Tuoren® videolaryngoscope provides faster and easier intubation, suggesting its potential as a reliable and cost-effective alternative in clinical practice. Further large-scale studies are warranted to confirm these findings across varied clinical scenarios.

## Introduction

Laryngoscopy is a fundamental procedure in the practice of anesthesiology, essential for several critical tasks, including controlled ventilation, airway protection, removal of secretions, lung isolation, and the administration of anesthetic gases. The technique's history dates back to 1878, when Macewen first introduced tracheal intubation [1]. Since then, the field has seen many innovations aimed at enhancing the safety and efficiency of the procedure. Video laryngoscopy offers advantages such as improved glottic exposure, higher success rates, and reduced instances of difficult intubation, making it an integral component of modern anesthesia practice [2]. Effective intubation techniques and tools are crucial for ensuring patient safety, particularly in emergency situations where swift and accurate airway management can be lifesaving. By refining laryngoscopic methods and devices, anaesthesiologists can reduce the incidence of airway trauma, hypoxia, and other potentially severe complications. The ongoing development and assessment of intubation tools, such as different laryngoscope blades and video-assisted devices, are vital for enhancing the efficiency and reliability of this critical procedure, ultimately leading to better patient care and outcomes.

The King Vision® videolaryngoscope (Ambu A/S, Ballerup, Denmark) was developed in 2013 and introduced into clinical use in 2014. It has a reusable handle with a screen and disposable L-shaped blades that provide a clear view of the larynx without needing airway alignment. It also includes a guiding channel that helps pass the endotracheal tube, making it easier for less experienced users [3]. The Tuoren® videolaryngoscope (Tuoren Medical Group, Henan, China) is a relatively new videolaryngoscope. It is a handheld device used for more precise intubation. It is a rigid video laryngoscope with a Macintosh blade and a detachable LED screen.

Studies have suggested its efficacy in providing a clear glottic view, yet comprehensive comparative analyses with other devices, especially the widely utilized King Vision®, are limited. We conducted a comprehensive comparison of the ease of intubation between the Tuoren® and King Vision® videolaryngoscopes by systematically analyzing parameters such as glottic visualization, time efficiency, success rates, and ease of intubation.

## Materials and methods

Study design

A prospective, randomized, observational, single-blind trial was conducted on 98 participants at the Department of Anaesthesiology and Critical Care at Tata Main Hospital, a tertiary-care center in Jamshedpur, Jharkhand, India, from August 2022 to April 2024.

Ethical consideration

The study received approval from the Institutional Ethics Committee of Tata Main Hospital, Jamshedpur, India (approval number: TMH/IEC/JUNE/010/2022). Written informed consent was obtained from all participants, and patient confidentiality was strictly maintained.

Study participants

After Institutional Ethics Committee approval and informed consent, the study was conducted on patients undergoing elective surgical procedures under general anesthesia.

Patients with cervical spine pathology/instability, restricted mouth opening (interincisor gap <3 cm), poor dental condition, burn contracture, patients with obvious airway anatomical deformities, and pregnant women were excluded from the study.

Methodology

Before surgery, an independent anaesthesiologist evaluated all patients for factors like age, sex, American Society of Anesthesiologists (ASA) physical status, weight, and height. Airway assessment included predictive indices for difficult intubation based on the El-Ganzouri Risk Index (EGRI), which scores from 0 to 12. A score < 4 predicts easy intubation, while a score of 4 or higher indicates potential difficulty. Subjects were categorized for difficult intubation propensity using the EGRI score. In our study, we only included patients with an EGRI score of 4 or less [4].

The participants were allocated into two groups of 49 each based on the type of videolaryngoscope used: Group I using the Tuoren® device and Group II using the King Vision® device. Randomization was performed using a computer-generated random number sequence with the sealed-envelope method.

After the patients were transferred to the operating room, standard ASA monitors were applied, and baseline parameters were recorded. The patient’s intravenous (IV) access was secured with an IV cannula of appropriate size under aseptic precautions, and 0.9% normal saline was started. Patients were preoxygenated with 100% oxygen for three minutes. A standard anesthetic induction regimen consisting of injection of midazolam (1 mg), injection of fentanyl (2 mcg/kg), and injection of propofol (2 mg/kg) was used in all patients. After confirming adequate bag-mask ventilation (BMV), intravenous vecuronium 0.1 mg/kg was administered, and ventilation was continued with oxygen plus isoflurane (1%-2%) for four minutes. If BMV was inadequate, the subject was excluded from the study, and further intervention in these patients followed standard difficult airway management guidelines.

Laryngoscopy (laryngoscope size 4) was done according to the assigned group using either the Tuoren® videolaryngoscope or the King Vision® videolaryngoscope by a designated anesthesiologist (> 5 years of experience & experience of > 30 intubations with each device). A total of three attempts were permitted for achieving successful intubation with an appropriately sized endotracheal tube. Patients were excluded from the study if intubation was unsuccessful after three attempts or if the time to successful intubation exceeded 120 seconds. Time taken for intubation, hemodynamic parameters, complications such as a fall in SpO₂ < 95% and airway trauma (bruises, lacerations, bleeding, dental damage, etc.), esophageal intubation, and number of attempts were observed. The performing anesthesiologists rated the overall intubation conditions using a satisfaction score on a scale of 0 to 4. 0: Failure (intubation not possible), 1: Poor (required an additional adjunct other than the videolaryngoscopy); 2: Fair (needed an extra adjunct and took more than 90 seconds); 3: Moderate (needed an extra adjunct but completed within 90 seconds); 4: Good (successful intubation on the first or second attempt within 90 seconds, without additional adjuncts). Comparison of ease of intubation score was our primary objective [5].

The primary investigator also recorded the Percentage of Glottic Opening (POGO) and the Cormack-Lehane grading of each subject [6-7]. The pertinent airway parameters observed during the study include ease of intubation score, laryngeal views obtained with laryngoscopy (modified Cormack-Lehane scale and POGO), ease of intubation on a four-point scale, number of attempts at laryngoscopy, and total laryngoscopy duration (in seconds). Hemodynamic parameters were recorded at predefined time points: before laryngoscopy (BL), post-induction, pre-intubation, and at one, two, three, five, and 10 minutes after laryngoscopy. Any complications during the procedure of laryngoscopy and intubation, such as trauma to lips, bleeding, bruises, laceration, dental damage, desaturation, and esophageal intubation, were also recorded.

Statistical analysis

Data recording was done using Microsoft Excel (Microsoft Corp, Redmond, WA, USA) and was analyzed using the IBM SPSS software for Windows, version 21.0. (IBM Corp., Armonk, NY, USA).

## Results

Study findings reveal no significant differences between the Tuoren® group and King Vision® group in patients with regard to demographic characteristics such as age (p=0.647), sex (p=0.521), weight (p=0.275), height (p=0.905), body mass index (p=0.595), and ASA status (p=0.061) (Table 1). Though individually the thyromental distance (p = 0.022) and Mallampati score (p = 0.014) were different between the two groups, the consolidated EGRI score (2.2 ± 0.84 vs. 2.0 ± 0.42) (p = 0.295) between the two groups was also comparable (Table 2).

**Table 1:** Comparison of baseline patient characteristics between the Tuoren® and King Vision® groups Values are presented as Mean (M) ± SD unless otherwise indicated. Continuous variables were compared using independent-samples t-tests, and categorical variables were compared using Pearson's chi-square (χ²) tests. Percentages are column percentages. Statistical significance was set at p < .05. ASA = American Society of Anaesthesiologists

Characteristic (n=98)	Tuoren® (n = 49)	King Vision® (n = 49)	Statistic	p
Age (years), M ± SD	51.3 ± 15.2	49.9 ± 15.6	t(96) = −0.46	.647
Weight (kg), M ± SD	65.7 ± 9.3	67.6 ± 7.9	t(96) = 1.10	.275
Height (m), M ± SD	1.60 ± 0.06	1.60 ± 0.03	t(96) = 1.40	.257
Body mass index (kg/m²), M ± SD	25.5 ± 3.3	25.8 ± 2.9	t(96) = 0.53	.595
Sex, n (%)				
Female	34 (69.4)	31 (63.3)	χ²(1) = 0.40	.521
Male	15 (30.6)	18 (36.7)		
ASA physical status, n (%)				
I	7 (14.3)	17 (34.7)	χ²(2) = 5.60	.061
II	33 (67.3)	26 (53.1)		
III	9 (18.4)	6 (12.2)		

**Table 2:** Airway assessment (El-Ganzouri Risk Index score) Values are presented as n (%) or Mean (M) ± SD, as appropriate. Categorical variables were compared using Pearson's chi-square (χ²) test or Fisher's exact test, as appropriate. Continuous variables were compared using the independent-samples t test. Percentages are reported within study groups. Statistical significance was set at p < .05.

Characteristic (n=98)	Tuoren® (n = 49)	King Vision® (n = 49)	Statistic	p
Mouth				
> 4 cm	48 (98.0)	48 (98.0)	χ²(1) = 0.40	> .999
< 4 cm	1 (2.0)	1 (2.0)		
Thyromental distance, n (%)				
> 6.5 cm	34 (69.4)	44 (89.8)	χ²(1) = 6.28	.022
6.0–6.5 cm	15 (30.6)	5 (10.2)		
< 6.0 cm	0 (0.0)	0 (0.0)		
Modified Mallampati score, n (%)				
Grade I	10 (20.4)	3 (6.1)	χ²(2) = 7.98	.014
Grade II	36 (73.5)	46 (93.9)		
Grade III–IV	3 (6.1)	0 (0.0)		
Neck movement				
> 90°	48 (98.0)	49 (100.0)	χ²(1) = 1.01	> .999
90°	1 (2.0)	0 (0.0)		
< 90°	0 (0.0)	0 (0.0)		
El-Ganzouri Risk Index total score, M ± SD	2.2 ± 0.84	2.0 ± 0.42	t(96) = −1.10	.292

The percentage of successful intubations in the first and second attempts was comparable between the groups (p=0.749). The mean intubation time was significantly shorter in the Tuoren® group compared with the King Vision® group (64.6±28.8 vs. 79.6±13.2, p<0.001). The Tuoren® videolaryngoscope had ease of intubation scores of 4 and 3 in 38.8% of patients each. The King Vision® videolaryngoscope had an ease of intubation score of 4 (good) and 3 (moderate) in 6.1% and 69.4% of patients, respectively. The overall ease of intubation score was significantly higher for the Tuoren® videolaryngoscope group than the King Vision® videolaryngoscope group, with p=0.0011 (median 3 (IQR 3-4) vs. median 3 (IQR 3-3)) (Table 3). The King Vision® group offered a better glottic view compared with the Tuoren® group according to the modified Cormack and Lehane grading system (p = 0.002) (Table 4), although the percentage of glottic opening was comparable (p = 0.64). There were no significant differences between the groups in hemodynamic parameters at baseline, during intubation, or after the procedure. The mean intubation time was significantly shorter in the Tuoren® group as compared to the King Vision® group (64.6 ± 28.8 vs. 79.6 ± 13.2; p < 0.001).

**Table 3:** Comparison of ease of intubation scores between the Tuoren® and King Vision® groups Values are presented as n (%) unless otherwise indicated. Group comparisons for ease of intubation score distribution were performed using Fisher's exact test. Statistical significance was defined as p < .05.

Ease of Intubation Score (n=98)	Tuoren® (n = 49)	King Vision® (n = 49)	Statistic	p
4 (Good), n (%)	19 (38.8)	3 (6.1)	χ²(3) = 16.80	< .001
3 (Moderate), n (%)	19 (38.8)	34 (69.4)		
2 (Fair), n (%)	11 (22.4)	11 (22.4)		
1 (Poor), n (%)	0 (0.0)	1 (2.1)		
0 (Failure), n (%)	0 (0.0)	0 (0.0)		
Median (IQR)	3 (3–4)	3 (3–3)		

**Table 4:** Comparison of modified Cormack–Lehane glottic view grades between the Tuoren® and King Vision® groups Values are presented as n (%). Group comparisons were performed using Fisher's exact test because of sparse cell frequencies. Percentages are column percentages. Statistical significance was defined as p < .05.

Modified Cormack–Lehane Grade (n=98)	Tuoren® (n = 49)	King Vision® (n = 49)	Statistic	p
1, n (%)	27 (55.1)	29 (59.2)	χ²(4) = 6.51	.002
2, n (%)	2 (4.1)	0 (0.0)		
2a, n (%)	16 (32.7)	20 (40.8)		
2b, n (%)	3 (6.1)	0 (0.0)		
4, n (%)	1 (2.0)	0 (0.0)		

## Discussion

Our study compared two videolaryngoscope blade designs, the King Vision® and Tuoren® videolaryngoscopes, for intubation in predicted easy airways by anesthesiologists. Study results revealed that as far as ease of intubation is concerned, the Tuoren® videolaryngoscope performed better than the King Vision® videolaryngoscope. The Tuoren® videolaryngoscope demonstrated a reduced intubation time and greater ease. Our findings were in stark contrast to the study published by Gupta et al., who concluded that better intubating conditions were seen with King Vision® as compared to the Tuoren® videolaryngoscope [3]. However, their study was performed on airway mannequins in a simulated COVID-19 scenario, whereas our study was conducted in live patients posted for elective surgery by a single experienced operator. This makes our study a more pragmatic evaluation of the performance of these devices in a real-world clinical setting.

Laryngeal view

Our study results imply that the laryngeal view was significantly better with the King Vision® videolaryngoscope when compared with the Tuoren® videolaryngoscope. This is in agreement with Gupta et al., who employed Cook’s modification of the Cormack-Lehane grading system in their research to assess ease of intubation and laryngeal view with the Tuoren® and King Vision® videolaryngoscopes. They observed that the King Vision® channeled videolaryngoscope provided better glottic visualization and higher first-attempt success rates in a simulated COVID-19 scenario using mannequins [3].

Ramesh et al. compared the King Vision® (non-channeled blade) and Tuoren® videolaryngoscopes in patients with cervical spine immobilization and reported faster glottic visualization with the Tuoren® device. This finding is in agreement with our study, which also demonstrated shorter glottic visualization times with the Tuoren® videolaryngoscope. However, the study populations differed, as their study involved patients with simulated difficult airways, whereas our study included patients with normal airways [8].

A study by Rajan et al. found that glottic visualization during nasotracheal intubation was comparable between the C-MAC and Tuoren® videolaryngoscopes. In contrast, our study demonstrated significantly better glottic visualization with the King Vision® videolaryngoscope compared to the Tuoren® videolaryngoscope in patients undergoing elective orotracheal intubation with normal airways. The difference in findings may be attributed to variations in the type of videolaryngoscopes used, the route of intubation (nasotracheal versus orotracheal), and the study population [9].

Laryngoscopy duration

Group B-King Vision® had a significantly higher mean duration of laryngoscopy as compared to Group A (Tuoren®; p < 0.001). This finding is in contrast to the study reported by Gupta et al., who found that the time taken for laryngoscopy was significantly lower with the King Vision® videolaryngoscope compared to the Tuoren® videolaryngoscope [3]. A possible explanation can be the fact that our study was conducted on live human subjects instead of airway mannequins. These mannequins cannot fully reproduce real human airway characteristics such as natural anatomical variations, secretions, and reflexes. These real-world factors can influence the laryngoscopy experience and thereby alter the procedure time, as witnessed in our study.

In a study by Bhagat et al., the Tuoren® videolaryngoscope was compared with the Macintosh laryngoscope in patients with normal airways. The authors reported that the Tuoren® Video Laryngoscope provided significantly shorter intubation time and required fewer optimization maneuvers than the Macintosh laryngoscope. These findings are consistent with the results of our study, where the Tuoren® videolaryngoscope also demonstrated superior intubating conditions compared with the King Vision® videolaryngoscope. However, the comparator device differed between the two studies, with the previous study comparing Tuoren® with the conventional Macintosh laryngoscope, whereas our study compared two video laryngoscopes [10].

Ease of intubation

Although the laryngeal view was better with the King Vision® videolaryngoscope in our study, the ease of intubation was much better with the Tuoren® videolaryngoscope.

This seeming dichotomy can be explained by the fact that the design of the Tuoren® videolaryngoscope allows better amalgamation of the virtual line of sight with the actual process of intubation. It is a known fact that while using a videolaryngoscope, it has been established that the view of the glottis inlet is improved, but this does not always translate to similar intubating conditions. Future studies are recommended to validate our results and investigate these devices in more challenging, difficult airway scenarios and diverse clinical settings and patient populations.

This study was conducted only in patients with anticipated normal airways (EGRI score < 4); therefore, the findings cannot be generalized to patients with difficult airways. Additionally, the study evaluated videolaryngoscope performance only during elective orotracheal intubation under controlled operating room conditions, limiting its applicability to other clinical scenarios such as nasotracheal intubation, cervical spine immobilization, and emergency airway management.

## Conclusions

In this prospective randomized study, both the Tuoren® and King Vision® videolaryngoscopes were effective for endotracheal intubation in patients with anticipated normal airways undergoing elective surgery. The King Vision® videolaryngoscope provided better glottic visualization, whereas the Tuoren® videolaryngoscope was associated with easier intubation and a shorter intubation time. The success rate, hemodynamic responses, and complication rates were comparable between the two devices.

These findings suggest that although both videolaryngoscopes are suitable for routine airway management, the Tuoren® videolaryngoscope may offer practical advantages in terms of ease and speed of intubation in the studied population. As this study was conducted at a single center and included only patients with anticipated normal airways, the results should be interpreted within this context. Further multicenter studies involving larger and more diverse patient populations, including those with difficult airways and emergency settings, are recommended to validate these findings.
